# Long non-conding RNA LOXL1-AS1 sponges miR-589-5p to up-regulate CBX5 expression in renal cell carcinoma

**DOI:** 10.1042/BSR20200212

**Published:** 2020-11-13

**Authors:** Chunlei Wu, Jiange Zhang

**Affiliations:** 1 Department of Urology, The First Affiliated Hospital of Xinxiang Medical University, Weihui 453100, Henan, China; 2 Department of Urology, The Second Affiliated Hospital of Guangxi Medical University, Nanning 530000, Guangxi, China

**Keywords:** CBX5, LOXL1-AS1, miR-589-5p, renal cell carcinoma

## Abstract

Background: Renal cell carcinoma (RCC) is a common malignant tumor that seriously endangers people’s health. In recent years, long non-coding RNAs (lncRNAs) have been discovered to play vital roles in diverse cancers, including RCC. LncRNA lysyl oxidase like 1 antisense RNA 1 (LOXL1-AS1) has been found to exert carcinogenic functions in several cancers, but its role and mechanism in RCC have not been investigated.

Methods: qRT-PCR was utilized for testing RNA expression and Western blot for protein expression in RCC tissues or cells. Then, we assessed cell function by conducting a series of functional experiments, such as 5-ethynyl-2′-deoxyuridine staining, colony formation, flow cytometry, JC-1, Western blot and transwell migration experiments. Following, RNA immunoprecipitation, pull down and luciferase reporter experiments were carried out to explore the regulatory mechanisms of LOXL1-AS1 in RCC.

Results: LOXL1-AS1 was highly expressed in RCC tissues and cells. Moreover, knockdown of LOXL1-AS1 hampered RCC cell proliferation and migration. Importantly, miR-589-5p that was lowly expressed and worked as a tumor-inhibitor in RCC was found to bind with LOXL1-AS1. Furthermore, chromobox 5 (CBX5) targeted by miR-589-5p could expedite cell proliferation and migration in RCC. Finally, overexpressed CBX5 or inhibited miR-589-5p reversed the repressive impacts of silenced LOXL1-AS1 on RCC malignant phenotypes.

Conclusions: LncRNA LOXL1-AS1 sequestered miR-589-5p to augment CBX5 expression in RCC cells, opening a new way for potential development in RCC treatment.

## Introduction

Renal cell carcinoma (RCC) is a common malignant tumor originating from renal tubular epithelial cells [1]. In recent years, the incidence of RCC is increasing [2]. Although the survival rate of patients with early RCC is increasing with the continuous development of current treatment methods, the treatment effect is not ideal for those with advanced RCC [3]. However, a lot of RCC patients have developed to middle or even late stage when diagnosed due to limited symptoms in early stage [4]. The prognosis of RCC patients in the middle and late stage is extremely poor [5]. Thus, in-depth understanding of the pathogenesis will be helpful to explore more effective treatment methods of RCC.

Long non-coding RNAs (lncRNAs) are members of non-coding RNAs family. Their length are more than 200 nucleotides and they possess no or limited capability of coding proteins [6]. In recent years, increasing evidence has indicated that lncRNAs take part in the process of biological activities like cell proliferation and cell apoptosis [7–9]. Importantly, lncRNAs have also been confirmed to serve as pivotal regulators in assorted diseases, including human cancers [10–12]. Moreover, the ceRNA (competing endogenous RNA) function of lncRNAs by sponging miRNAs to regulate mRNA expression has been largely suggested to be associated with cancer development [13–15]. For example, SNHG8 expedites the progression of hepatocellular carcinoma via sponging miR-149-5p [16]. XIST can exert the antitumor effect in breast cancer through miR-155/CDX1 axis [17]. Lysyl oxidase like 1 antisense RNA 1 (LOXL1-AS1) is a novel lncRNA that has been confirmed as a tumor-facilitator in several cancers. For example, LOXL1-AS1 can accelerate cell growth in medulloblastoma through activating PI3K/AKT pathway [18]. In addition, LOXL1-AS1 has also reported to function in prostate cancer via miR-541-3p/CCND1 axis [19]. Moreover, LOXL1-AS1 can serve as a sponge for miR-324-3p to expedite cholangiocarcinoma progression [20]. Nevertheless, the specific function and mechanism of LOXL1-AS1 in RCC have not been elaborated at present.

Thus, the central purpose of our work was to investigate the function and probable mechanism of LOXL1-AS1 in RCC, which may offer new ideas for RCC treatment.

## Materials and methods

### Tissues collection

A total of 60 pairs of RCC samples (cancerous and matched non-cancerous ones) were surgically acquired from RCC patients at the First Affiliated Hospital of Xinxiang Medical University. Samples were preserved at −80°C after quick freezing by liquid nitrogen. All patients without other treatments before operation signed the written informed consents prior to the present study. The approval numbered 2020190 from the Ethics Committee of the First Affiliated Hospital of Xinxiang Medical University supported the present study.

### Cell lines

Human embryonic kidney cell line (HEK-293) and human RCC cell lines (786-O, A-498, 769-P) were available from American Type Culture Collection (ATCC; Manassas, VA, U.S.A.). All were incubated with 10% fetal bovine serum (FBS) and 1% Pen/Strep mixture in Dulbecco’s Modified Eagle Medium (DMEM) in a humidified incubator containing 5% CO_2_ at 37°C, as requested by supplier (Invitrogen, Carlsbad, CA).

### Total RNA extraction and quantitative real-time polymerase chain reaction (qRT-PCR)

Total RNA was extracted from RCC tissues or cells in line with the instruction of Trizol reagent (Invitrogen). To examine gene expression, complementary DNA (cDNA) synthesis was completed using PrimeScript™ RT Reagent Kit (Takara, Shiga, Japan), and then SYBR Premix Ex Taq II (Takara) was used for qRT-PCR. Fold-change was calculated by the 2^−ΔΔCt^ method. House-keeping genes GAPDH (glyceraldehyde-3-phosphate dehydrogenase) and U6 snRNA were appropriately used for standardization.

### Plasmid transfection

The short hairpin RNAs (shRNAs) designed for LOXL1-AS1 or CBX5 by Genepharma (Guangzhou, China) were separately transfected into A-498 and 769-P cells by use of Lipofectamine 2000 (Invitrogen). Besides, the pcDNA3.1-CBX5 and the empty control pcDNA3.1-NC, miR-589-5p mimics and NC-mimics, miR-589-5p inhibitor and NC inhibitor were all produced by RiboBio (Guangzhou, China). The plasmid transfection was performed for 48 h, and then cells were reaped and collected for following use.

### 5-Ethynyl-2′-deoxyuridine staining assay

Cells in 96-well plates (5 × 10^4^/well) were incubated with 5-ethynyl-2′-deoxyuridine (EdU) medium diluent, and then fixed by 4% paraformaldehyde for 30 min. EdU staining assay reagent was produced by RiboBio for detecting cell proliferation. Cell samples were observed by fluorescence microscope (Olympus, Tokyo, Japan) after 4**′**,6-diamidino-2-phenylindole (DAPI) staining for nuclear detection.

### Colony formation assay

Clonogenic cells in six-well plates with 500 cells per well were incubated for 14 days. Colonies were then counted after fixing by 70% ethanol and staining in 0.5% Crystal Violet.

### Flow cytometry analysis

Annexin V-FITC (fluorescein isothiocyanate)/PI (Propidium Iodide) Apoptosis kit (BD Biosciences, San Jose, CA) was used for examining cell apoptosis as per user’s manual. The collected cell samples from precooled phosphate buffer saline (PBS) were double-stained for 15 min in darkroom, and then analyzed by flow cytometer (BD Biosciences).

### JC-1 assay

This experiment was implemented by use of a JC-1 Assay Kit (Beyotime, Shanghai, China). Cell samples in culture plates were centrifuged for loading with the JC-1 dye, and then processed with assay buffer for 30 min. Detection of mitochondrial transmembrane potential (ΔΨm) was performed by fluorescent plate reader, followed by imaging under fluorescence microscope.

### Western blot

Total protein samples were extracted from A-498 and 769-P cells and separated by 12% sodium dodecyl sulfate-polyacrylamide gel electrophoresis (SDS-PAGE), followed by shifting to polyvinylidene fluoride (PVDF) membranes. After sealing by 5% nonfat milk, membranes were probed with primary antibodies specific to the loading control GAPDH (ab181602) and caspase 3 (ab197202), cleaved caspase 3 (ab2302), caspase 9 (ab219590), cleaved caspase 9 (ab2324), Bax (ab32503), Bcl-2 (ab32124), E-cadherin (ab40772), N-cadherin (ab18203) and MMP2 (ab97779) all night. After washing in tris-buffered saline Tween-20 (TBST), the secondary antibodies were added for 2 h of incubation. The protein bands were visualized by enhanced chemiluminescence (ECL) detection system (Santa Cruz Biotechnology, Santa Cruz, CA). All used antibodies were purchased from Abcam (Cambridge, MA).

### Transwell migration assay

Cell migration ability was assessed by 8-mm pore size Transwell chambers as required by provider (Corning, Corning, NY). Lower chamber was filled with complete medium. Cell samples suspended in serum-free medium were plated into upper chamber for 24 h of incubation. After that, cells migrating into the bottom were counted under microscope after being fixated and stained. Five fields were randomly selected for counting of migrated cells.

### Subcellular fraction

Cells (1 × 10^6^) reaped by precooled PBS were processed with cell fractionation buffer and cell disruption buffer, in succession. The cytoplasmic and nuclear fractions were isolated individually using the PARIS™ Kit (Invitrogen). LOXL1-AS1 content in both fractions was detected by qRT-PCR.

### Fluorescence *in situ* hybridization

The fluorescence-conjugated LOXL1-AS1 probe was constructed by RiboBio. Cell samples were hybridized with LOXL1-AS1 probe, counterstained by DAPI dye and finally observed using fluorescence microscope.

### RNA pull down assay

Cells were lysed via Radio Immunoprecipitation Assay (RIPA) lysis buffer, and then cell lysates were mixed with the biotin-labeled RNAs including Bio-NC, Bio-miR-589-5p-WT and Bio-miR-589-5p-Mut. Following the addition of magnetic beads, RNAs in the pulled down mixture were eluted for qRT-PCR analysis.

### Luciferase reporter assay

LOXL1-AS1 or CBX5 fragment covering wild-type (WT) or mutant (Mut) miR-589-5p target sites was individually inserted into pmirGLO luciferase reporter vector, which was then named as LOXL1-AS1-WT/Mut or CBX5-WT/Mut, respectively. The constructed vectors were co-transfected with miR-589-5p mimics or NC mimics into A-498 and 769-P cells by using Lipofectamine 2000. Luciferase Reporter Assay system (Promega, Madison, WI) was finally used for analysis of the luciferase activity after 48 h of transfection.

### RNA immunoprecipitation

Cells were lysed by RNA immunoprecipitation (RIP) lysis buffer, and then cell lysates were subjected to overnight incubation with magnetic beads conjugated with human Argonaute RISC catalytic component 2 (Ago2) antibody (Millipore, Billerica, MA, U.S.A.). Besides, the normal mouse IgG antibody acquired from Millipore served as the negative control. Relative RNA enrichment was examined by qRT-PCR.

### Statistical analysis

All experiments were conducted in triplicate. Results were given as the mean ± standard deviation (SD) and progressed by Prism 5.0 software (GraphPad Software, Inc., La Jolla, CA). Data were analyzed in form of Student’s *t*-test or one-way analysis of variance (ANOVA), with *P*<0.05 as the threshold of significant value.

## Results

### LOXL1-AS1 was up-regulated in RCC cells and silencing LOXL1-AS1 blocked RCC cell growth and migration

**Figure 1:**
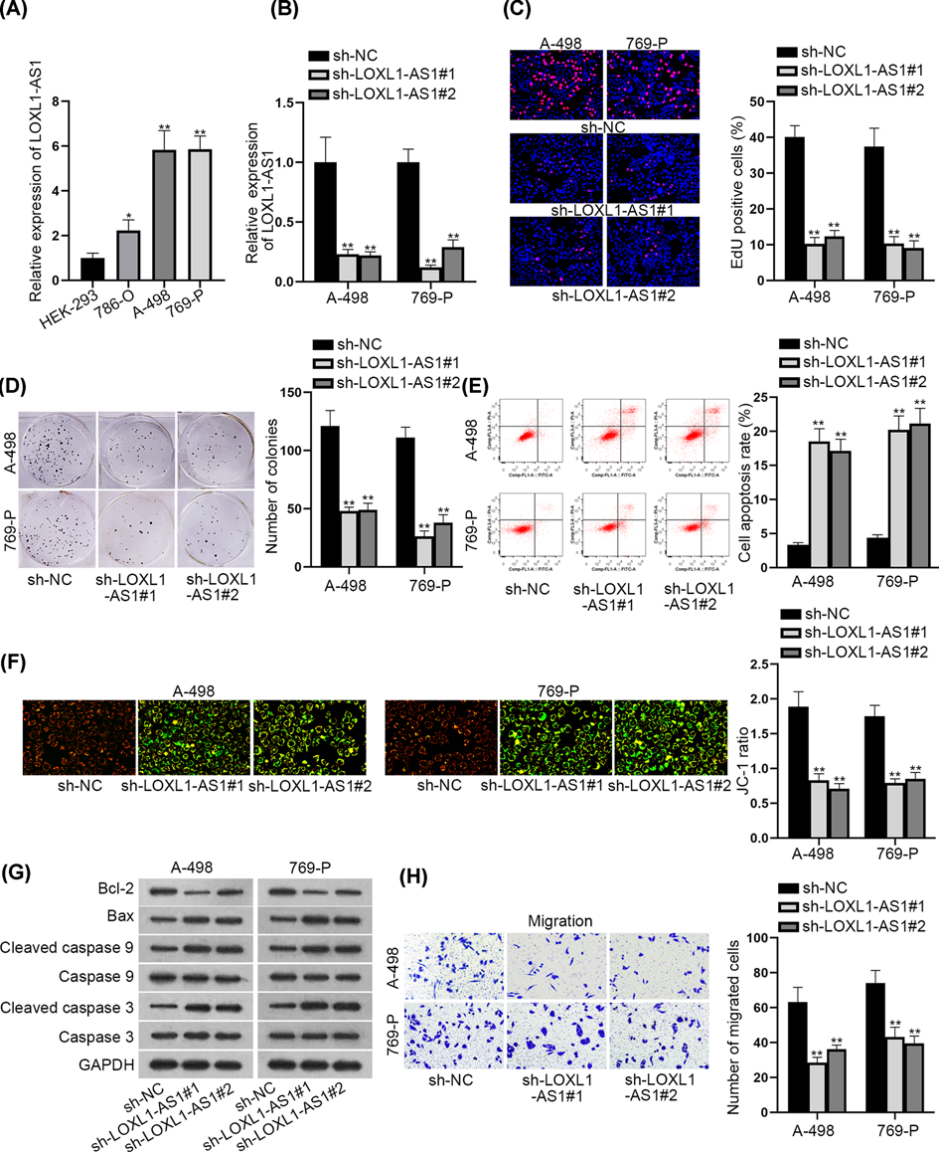
LOXL1-AS1 was up-regulated in RCC cells and LOXL1-AS1 facilitated RCC cell proliferation and migration (**A**) The expression of LOXL1-AS1 in RCC cells (786-O, A-498 and 769-P) was tested through qRT-PCR. (**B**) The depletion efficiency of LOXL1-AS1 in A-498 and 769-P cells was evaluated through qRT-PCR. (**C** and **D**) EdU staining and colony formation experiments were utilized to measure the proliferation of A-498 and 769-P cells after knockdown of LOXL1-AS1. (**E** and **F**) Flow cytometry and JC-1 experiments were adopted to test cell apoptosis when LOXL1-AS1 was silenced. (**G**) The changes on levels of apoptosis-related proteins were estimated by Western blot after silencing LOXL1-AS1. (**H**) Transwell experiments tested the influence of inhibiting LOXL1-AS1 on RCC cell migration; *P<0.05, **P<0.01.

A lot of researches indicated that the progression of cancers was associated with the dysregulation of lncRNAs [18,19]. Therefore, we wondered whether the expression of LOXL1-AS1 was altered in RCC compared with normal conditions. Interestingly, LOXL1-AS1 exhibited an elevated expression in RCC tissues relative to paired non-cancerous ones (Supplementary Figure S1A). Also, RCC cell lines, including 786-O, A-498 and 769-P cells, expressed higher levels of LOXL1-AS1 than the normal HEK-293 cells (Figure 1A). Then, we performed loss-of-function assays in A-498 and 769-P cells who owned highest LOXL1-AS1 level, with the knockdown efficiency of LOXL1-AS1 validated in Figure 1B. As a result, we discovered that the proportion of EdU positive cells and the number of colonies declined in face of LOXL1-AS1 inhibition (Figure 1C,D), indicating that cell proliferative capability was restrained when LOXL1-AS1 was silenced in A-498 and 769-P cells. Following, we could observe that the rate of apoptotic A-498 and 769-P cells was ascended under LOXL1-AS1 silence (Figure 1E). Similarly, JC-1 ratio was reduced, and the levels of pro-apoptosis proteins (Bax and cleaved caspase-3/9) was elevated while that of anti-apoptosis protein Bcl-2 declined in response to LOXL1-AS1 inhibition (Figure 1F,G). Moreover, the results of transwell assays depicted that cell migration was hampered due to the lack of LOXL1-AS1 (Figure 1H). Besides, such phenomenon was further testified by enhanced E-cadherin level and reduced levels of N-cadherin and MMP2 in these two RCC cells facing LOXL1-AS1 deficiency (Supplementary Figure S1B). Overall, LOXL1-AS1 was up-regulated in RCC cells and its suppression restrained RCC cell proliferation and migration.

### LOXL1-AS1 interacted with miR-589-5p and acted as its sponge

**Figure 2:**
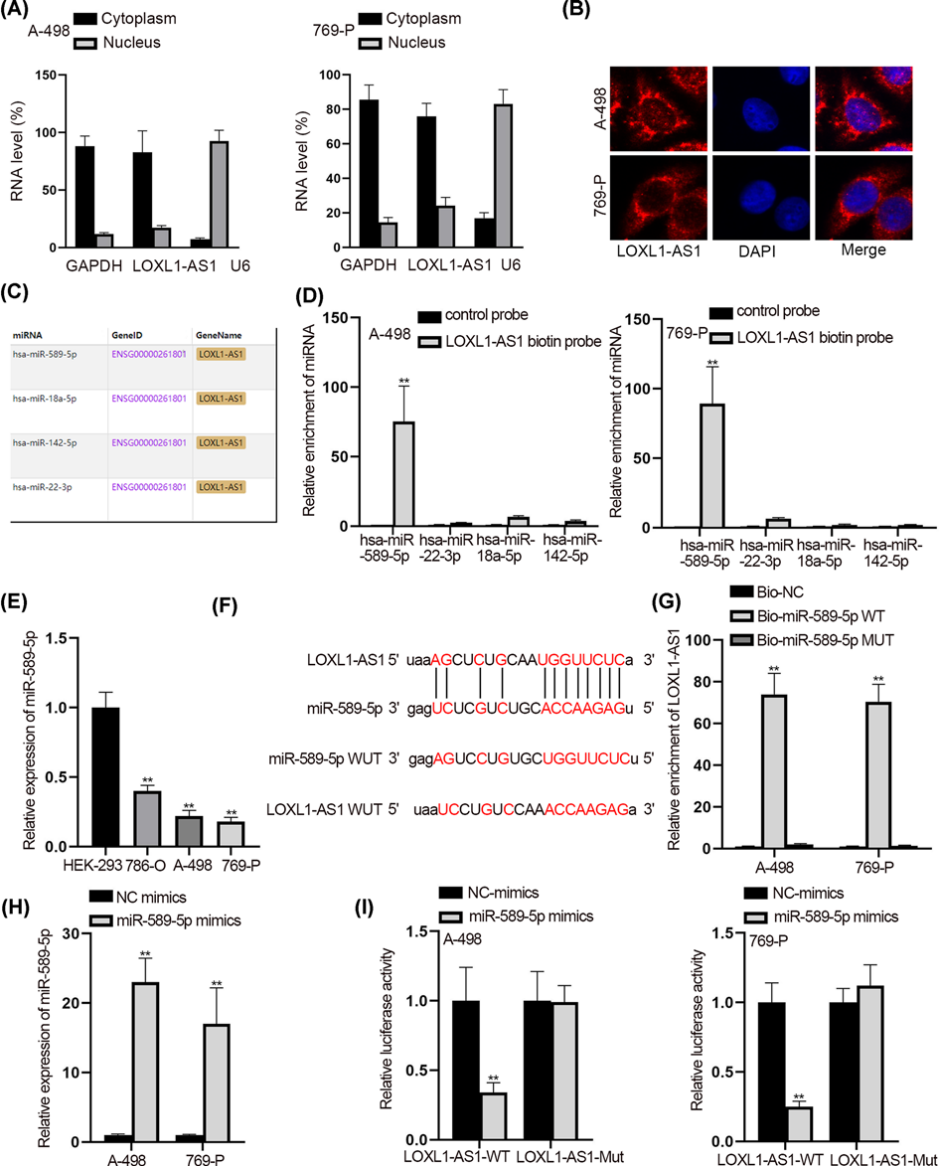
LOXL1-AS1 interacted with miR-589-5p and acted as its sponge (**A** and **B**) Subcellular fractionation and Fluorescence *in situ* hybridization (FISH) assays were utilized to detect the distribution of LOXL1-AS1 in A-498 and 769-P cells. (**C**) The possible miRNAs were discovered from starBase under the condition (Pan-Cancer ≥ 10). (**D**) RNA pull down experiment was utilized to detect the binding situation between above miRNAs and LOXL1-AS1. (**E**) The expression of miR-589-5p in RCC cells was tested through qRT-PCR. (**F**) The binding sites between LOXL1-AS1 and miR-589-5p were predicted by starBase. (**G**) RNA pull down experiment was implemented to verify the correlation between LOXL1-AS1 and miR-589-5p. (**H**) The overexpression efficiency of miR-589-5p was tested via qRT-PCR. (**I**) Luciferase reporter experiment was conducted to prove the interaction between LOXL1-AS1 and miR-589-5p; **P<0.01.

Thereafter, we planned to explore the downstream mechanism of LOXL1-AS1 in RCC. Extensive studies have confirmed that lncRNAs locating in the cytoplasm can be implicated in the ceRNA network [21,22]. Intriguingly, we unveiled the mainly cytoplasmic distribution of LOXL1-AS1 in both A-498 and 769-P cells (Figure 2A,B). Then starBase (http://starbase.sysu.edu.cn/index.php) was utilized to seek out the potential miRNAs binding with LOXL1-AS1. On the basis of the condition (Pan-Cancer ≥ 10), four miRNAs (miR-589-5p, miR-22-3p, miR-18a-5p and miR-142-5p) were discovered (Figure 2C). In order to select the eligible miRNAs in RCC, we conducted RNA pull down experiments. Results revealed that miR-589-5p was highly enriched in the pull down compounds of LOXL1-AS1 biotin probe, while other miRNAs were not (Figure 2D). Also, we discovered the low expression of miR-589-5p in RCC cells, extremely in A-498 and 769-P cells (Figure 2E). In addition, the decreased miR-589-5p level was found in RCC tissues in comparison with paired non-tumor ones (Supplementary Figure S1C). Then the binding sites between miR-589-5p and LOXL1-AS1 were provided by starBase, and the mutated sequences of miR-589-5p and LOXL1-AS1 (miR-589-5p-Mut and LOXL1-AS1-Mut) were also presented (Figure 2F). The outcomes of RNA pull down experiments disclosed the high enrichment of LOXL1-AS1 in Bio-miR-589-5p-WT group rather than other two groups (Figure 2G). More importantly, miR-589-5p elevation induced evident reduction on the luciferase activity of LOXL1-AS1-WT, but not on that of LOXL1-AS1-Mut (Figure 2H,I). Thereafter, we probed into the impact of miR-589-5p on the function of RCC cells. It was exhibited that enhanced expression of miR-589-5p led to restrained proliferation, accelerated apoptosis and obstructed migration in A-198 and 769-P cells (Supplementary Figure S1D–J). In sum, LOXL1-AS1 could directly interact with the tumor-suppressive miR-589-5p in RCC.

### CBX5 was the target of miR-589-5p in RCC

**Figure 3:**
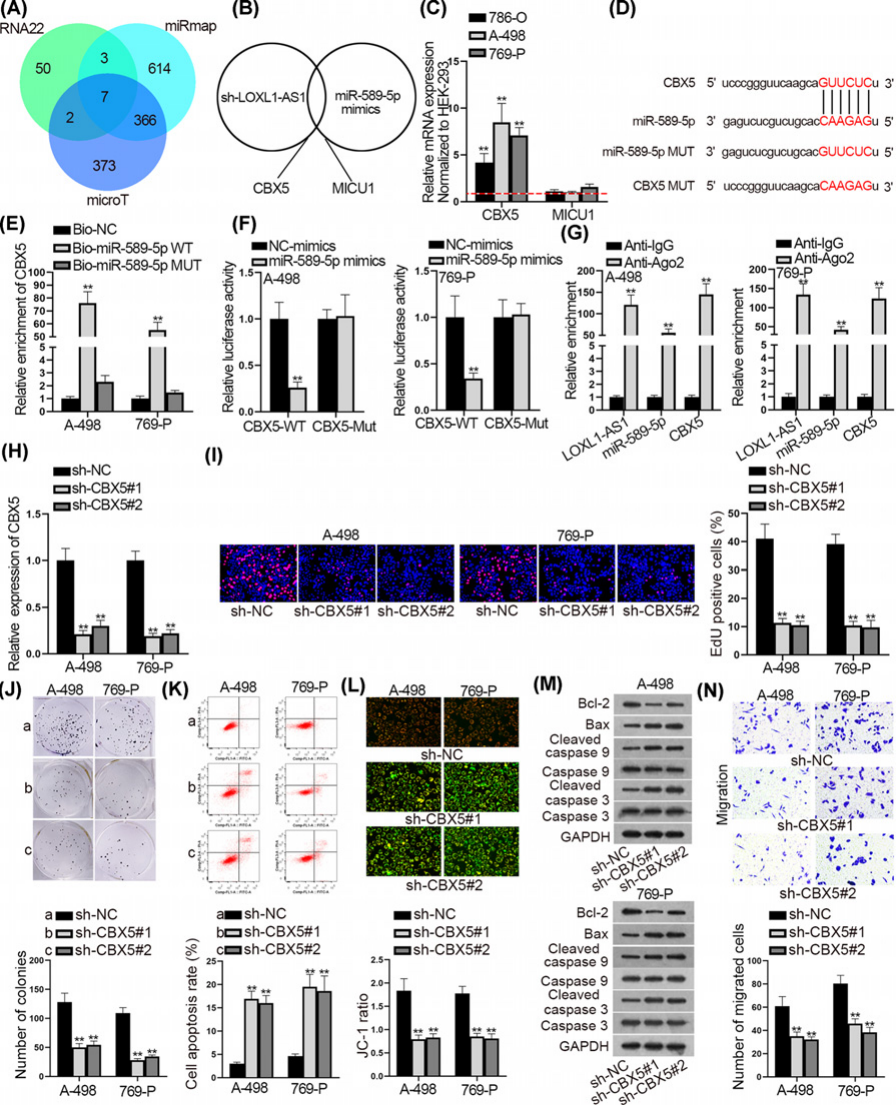
CBX5 was the target of miR-589-5p in RCC (**A**) StarBase was adopted to search the possible mRNAs that could bind with miR-589-5p. (**B**) The qRT-PCR experiment was utilized to test the expression of mRNAs when LOXL1-AS1 was inhibited and miR-589-5p was overexpressed. CBX5 and MICU1 were discovered to be down-regulated under silenced LOXL1-AS1 and overexpressed miR-589-5p. (**C**) The expression levels of CBX5 and MICU1 in RCC cells were detected by qRT-PCR. (**D**) The binding sites between CBX5 and miR-589-5p were displayed. (**E–G**) RNA pull down, luciferase reporter and RIP experiments were implemented to evaluate the interactions among LOXL1-AS1, miR-589-5p and CBX5. (**H**) The interference efficiency of CBX5 was measured by qRT-PCR. (**I** and **J**) EdU staining and colony formation experiments were utilized to test cell proliferation when CBX5 was inhibited. (**K–M**) Flow cytometry, JC-1 and Western blot experiments were implemented to estimate cell apoptosis after silencing CBX5. (**N**) Transwell experiments were conducted to test the influence of inhibiting CBX5 on cell migration; **P<0.01.

In order to further investigate the downstream mechanism, we utilized starBase to predict the possible mRNA targets of miR-589-5p. Under the prediction of RNA22, microT and miRmap databases, seven candidates were found (Figure 3A). However, only two of them (CBX5 and MICU1) were discovered to be down-regulated by LOXL1-AS1 depletion and miR-589-5p overexpression in the meantime (Figure 3B). Further, we discovered that the expression of CBX5 (chromobox 5) was evidently boosted in RCC cells, while that of MICU1 was not (Figure 3C). Also, CBX5 was dramatically up-regulated in RCC specimens compared with para-carcinoma tissues (Supplementary Figure S2A). More importantly, we proofed that CBX5 expression was in proportion to LOXL1-AS1 level but inversely proportional to miR-589-5p level in these clinical samples (Supplementary Figure S2B). Thereafter, we sought out the binding sites between CBX5 and miR-589-5p through starBase (Figure 3D). As expected, we observed that CBX5 was enriched in Bio-miR-589-5p-WT group (Figure 3E). Besides, the luciferase activity of CBX5-WT was effectively reduced by miR-589-5p mimics (Figure 3F). Significantly, LOXL1-AS1, miR-589-5p and CBX5 were all captured by anti-Ago2 (Figure 3G), indicating their coexistence in RNA-induced silencing complexes (RISCs). Next, we conducted some functional experiments to detect the cellular functions of CBX5 in RCC. After knocking down the expression of CBX5 in A-498 and 769-P cells (Figure 3H), the proliferation was hampered as demonstrated by EdU and colony formation experiments (Figure 3I,J). On the contrary, we discovered that cell apoptosis was elevated by CBX5 deficiency (Figure 3K–M). In addition, cell migration was also dampened after silencing CBX5 (Figure 3N and Supplementary Figure S2C). Taken together, CBX5 was the target of miR-589-5p and its deficiency impeded malignant phenotypes in RCC cells.

### LOXL1-AS1 accelerated the malignancy in RCC by regulating miR-589-5p and CBX5

**Figure 4:**
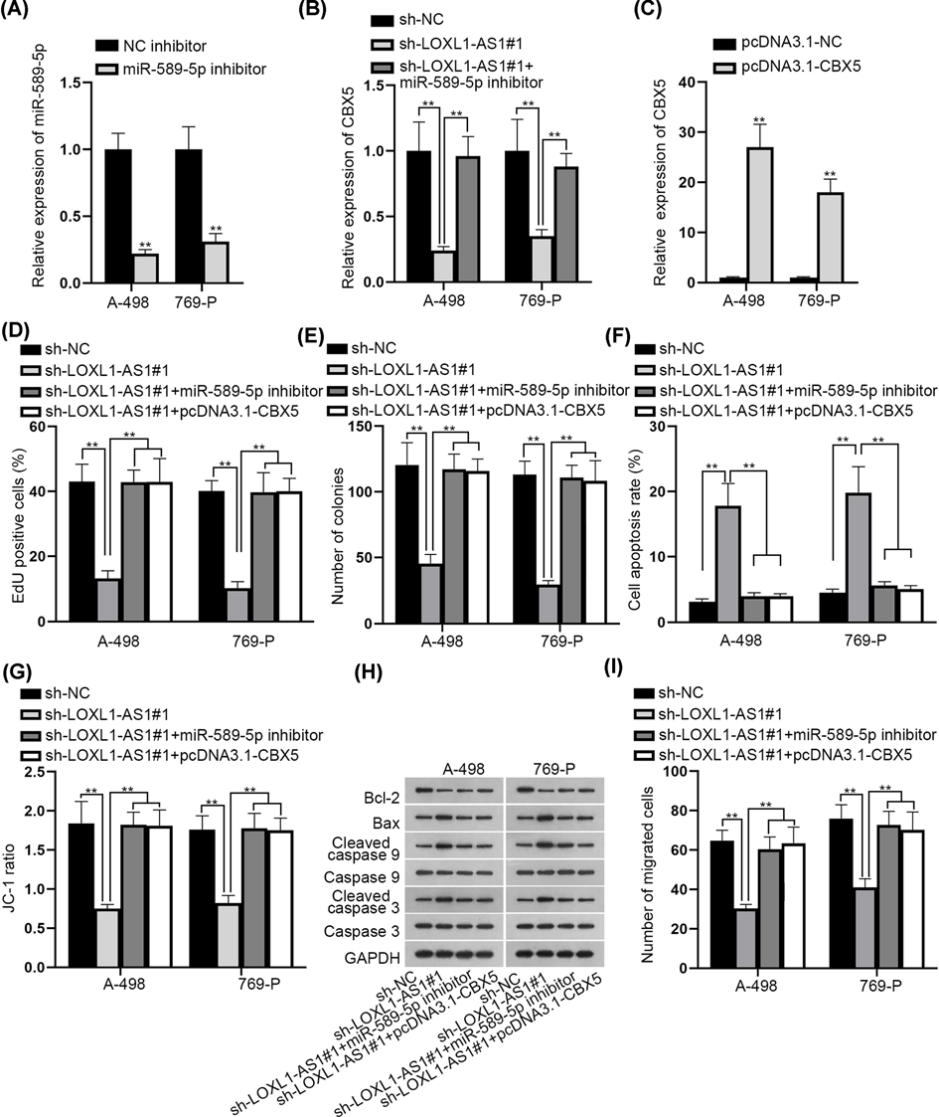
LOXL1-AS1 accelerated the progression of RCC by regulating miR-589-5p and CBX5 (**A**) The knockdown efficiency of miR-589-5p in A-498 and 769-P cells was tested by qRT-PCR. (**B**) The expression of CBX5 was tested by qRT-PCR when LOXL1-AS1 and miR-589-5p were inhibited. (**C**) qRT-PCR was utilized to test the overexpression efficiency of CBX5. (**D** and **E**) EdU staining and colony formation experiments were conducted to test cell proliferation in different groups. (**F–H**) Cell apoptosis was measured by flow cytometry, JC-1 and Western blot experiments in different groups. (**I**) The migration ability was evaluated through transwell experiments in different groups; **P<0.01.

At length, we implemented the rescue experiments to test whether LOXL1-AS1 affected RCC progression via miR-589-5p/CBX5 signaling. First of all, we tested that miR-589-5p was interfered in A-498 and 769-P cells by miR-589-5p inhibitor (Figure 4A). Resultantly, CBX5 expression declined by lacking LOXL1-AS1 was then reversed after miR-589-5p depletion (Figure 4B). Following, we overexpressed CBX5 in A-498 and 769-P cells, with the overexpression efficiency confirmed in Figure 4C. Next, we discovered that LOXL1-AS1 depletion-declined cell proliferative capability could be recovered by inhibiting miR-589-5p or overexpressing CBX5 (Figure 4D,E). Meanwhile, the stimulated cell apoptotic ability caused by silenced LOXL1-AS1 was reversed when miR-589-5p was inhibited or CBX5 was up-regulated (Figure 4F–H). Likewise, knockdown of miR-589-5p or up-regulation of CBX5 could offset the retarding effect of LOXL1-AS1 depletion on cell migration in RCC (Figure 4I and Supplementary Figure S2D). To sum up, LOXL1-AS1 accelerated the malignant behaviors in RCC via absorbing miR-589-5p and augmenting CBX5.

## Discussion

In recent years, lncRNAs have been widely concerned and researched because of their crucial biological functions in plenty of diseases, including human cancers. So far, a majority of lncRNAs have been revealed to take part in the progression of RCC. For example, MALAT1 accelerates RCC development by modulating Ezh2 through interacting with miR-205 [23]. PVT1 plays the oncogenic role in RCC via down-regulating miR-16-5p [24]. LOXL1-AS1 is a novel lncRNA that has not been studied in RCC. In our study, LOXL1-AS1 was discovered to be up-regulated in RCC tissues and cells. Additionally, silencing LOXL1-AS1 could dampen cell proliferation and migration. The whole experimental outcomes indicated that LOXL1-AS1 presented a carcinogenic property in RCC.

MicroRNAs (miRNAs) are small non-coding RNA molecules. Their length is about 22 nucleotides and they also exert vital functions in cancer. For example, miR-146b has been confirmed as a new biomarker and therapeutic target for human papillary thyroid cancer [25]. Moreover, miR-125a represses tumor growth in cervical cancer through targeting STAT3 [26]. Overexpression of miR-212 can repress tumorigenicity in hepatocellular carcinoma by inactivating Wnt/β-Catenin signaling pathway [27]. Importantly, numerous reports have recognized the importance of ceRNA network in human malignancies. For instance, PVT1/miR-190a-5p-miR-488-3p/MEF2C signaling was indicated to affect the development of human glioma [28]. In our research, we first confirmed the cellular localization of LOXL1-AS1 in cytoplasm. Then miR-589-5p was screened out as the downstream of LOXL1-AS1, and the low expression of miR-589-5p was discovered in RCC tissues and cells.

Further, we discovered CBX5 as the possible target of miR-589-5p. In previous research, it was disclosed that CBX5 accelerated cell proliferation and invasion in gastric cancer [28], indicating the CBX5 exerted an carcinogenic function in this disease. In our research, we unveiled that the expression of CBX5 was high in RCC tissues and cells. Furthermore, we verified that CBX5 was the target of miR-589-5p and that silencing CBX5 could block cell proliferation and migration in RCC cell lines. Finally, rescue experiments validated that LOXL1-AS1 elicited a tumor-facilitating function in RCC via miR-589-5p/CBX5-mediated manner.

In summary, LOXL1-AS1 up-regulated in RCC accelerated the malignant behaviors of RCC cells through regulating miR-589-5p/CBX5 pathway, which may offer useful theoretical basis for exploring the new effective therapeutic strategies for patients RCC.

## Supplementary Material

Supplementary Figure S1-S2

## Abbreviations

CBX5: chromobox 5
EdU: 5-Ethynyl-2′-deoxyuridine
LncRNA: long non-coding RNA
PBA: phosphate buffer saline
RCC: renal cell carcinoma
RISC: RNA-induced silencing complex
