# Supplementary material for: Long non-conding RNA LOXL1-AS1 sponges miR-589-5p to up-regulate CBX5 expression in renal cell carcinoma
Source: Biosci Rep. 2020 Nov 13;40(11):BSR20200212. doi: 10.1042/BSR20200212 (PMC7670581; doi:10.1042/BSR20200212)
Supplement: Supplementary Figure S1-S2 [file BSR-2020-0212_supp.pdf]

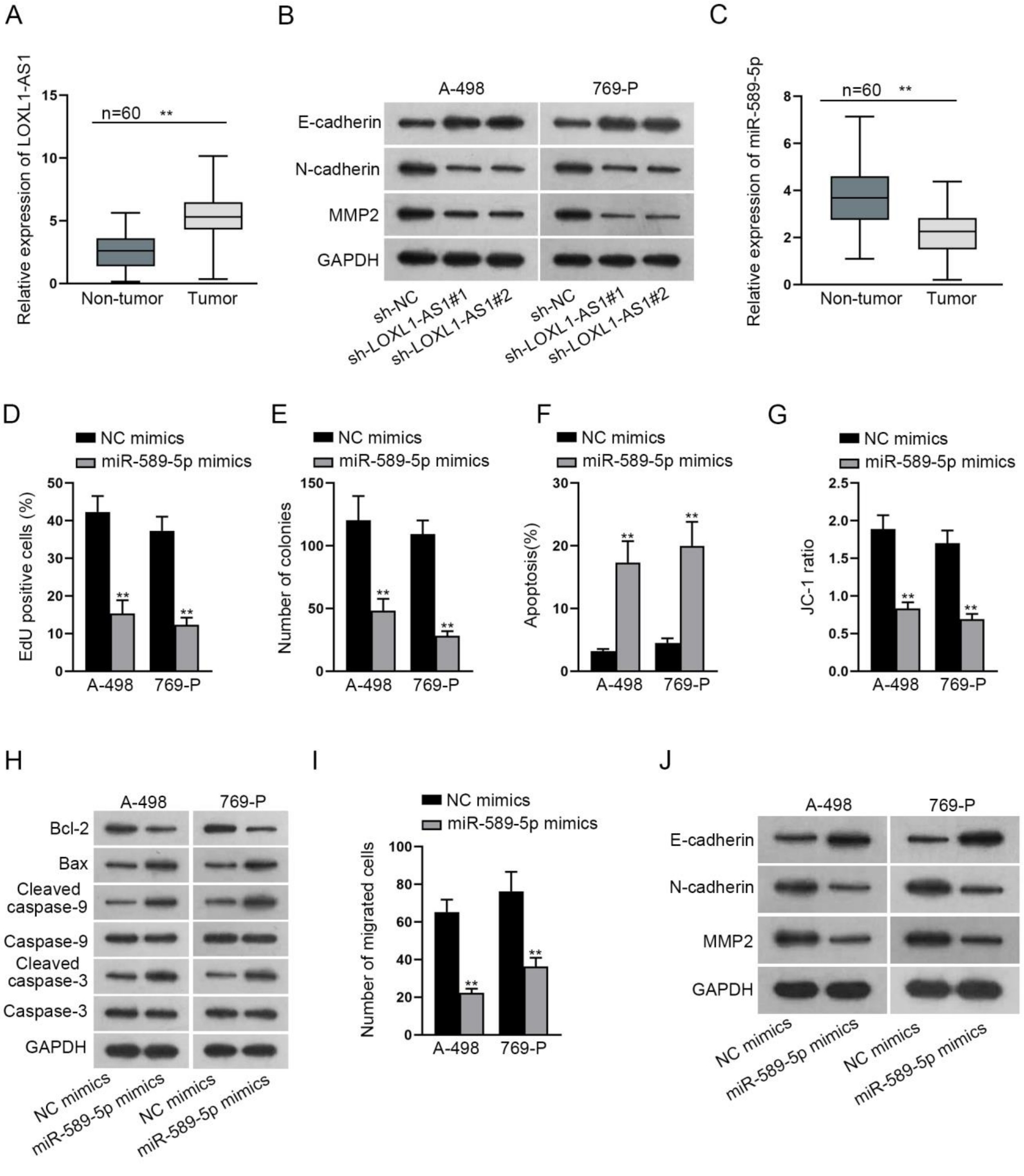
A
n=60 **
Relative expression of LOXL1-AS1
Non-tumor
Tumor
B
A-498
769-P
E-cadherin
N-cadherin
MMP2
GAPDH
sh-NC
sh-LOXL1-AS1#1
sh-LOXL1-AS1#2
C
n=60 **
Relative expression of miR-589-5p
Non-tumor
Tumor
D
NC mimics
miR-589-5p mimics
EdU positive cells (%)
A-498
769-P
E
Number of colonies
F
Apoptosis(%)
G
JC-1 ratio
H
Bcl-2
Bax
Cleaved caspase-9
Caspase-9
Cleaved caspase-3
Caspase-3
GAPDH
I
Number of migrated cells
J
E-cadherin
N-cadherin
MMP2
GAPDH

Supplementary figure 1 (A) Relative expression of LOXL1-AS1 in 60 pairs of RCC samples was analyzed by qRT-PCR. (B) The levels of migration-related proteins, including E-cadherin, N-cadherin and MMP2, were analyzed by western blot. (C) The level of miR-589-5p in 60 RCC tissues and paired non-tumor tissues was determined by qRT-PCR. (D-J) The impact of miR-589-5p overexpression on the proliferation, apoptosis and migration of A-498 and 789-P cells was assessed by EdU, colony formation and transwell assays, as well as related proteins determined by western blot analysis. **P < 0.01.

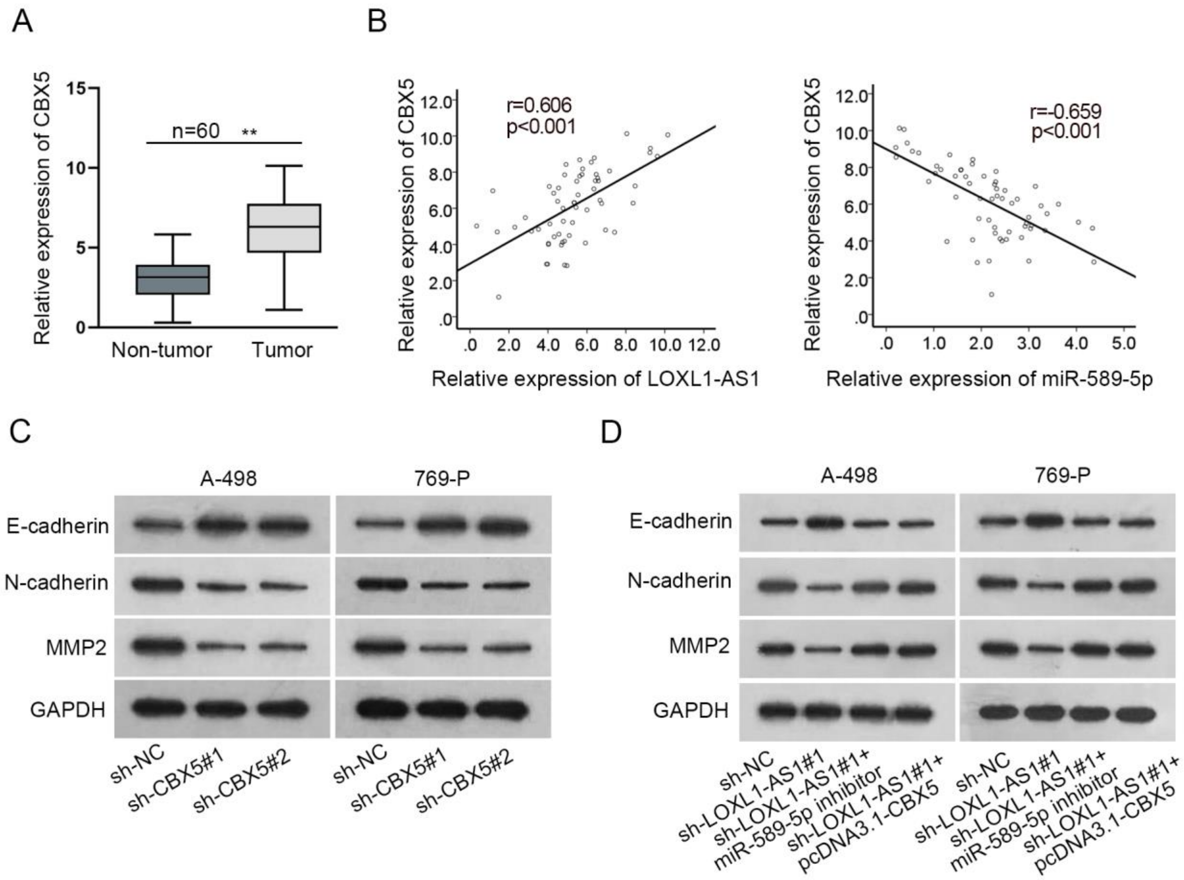
A
B
C
D
Relative expression of CBX5
n=60 **
Non-tumor
Tumor
r=0.606
p<0.001
Relative expression of LOXL1-AS1
r=-0.659
p<0.001
Relative expression of miR-589-5p
A-498
769-P
E-cadherin
N-cadherin
MMP2
GAPDH
sh-NC
sh-CBX5#1
sh-CBX5#2
sh-LOXL1-AS1#1
sh-LOXL1-AS1#1+ miR-589-5p inhibitor
sh-LOXL1-AS1#1+ pcDNA3.1-CBX5

Supplementary figure 2 (A) The expression of CBX5 in 60 RCC tissues and corresponding non-tumor tissues was estimated by qRT-PCR. (B) Pearson's correlation analysis validated the association of CBX5 expression with LOXL1-AS1 or miR-589-5p level in 60 RCC tissues. (C-D) The levels of E-cadherin, N-cadherin and MMP2 in A-498 and 789-P cells under different conditions were assessed by western blot.**P < 0.01.
